# Dose-dependent effects of mitoquinone on mitochondrial function and post-thaw quality of cryopreserved canine sperm

**DOI:** 10.14202/vetworld.2026.1273-1286

**Published:** 2026-03-23

**Authors:** Abbas Farshad, Emilia Diel, Axel Wehrend

**Affiliations:** Veterinary Clinic for Reproductive Medicine and Neonatology, Justus-Liebig-University of Giessen, 35392 Giessen, Germany

**Keywords:** canine sperm cryopreservation, mitoquinone, mitochondrial function, oxidative stress, post-thaw sperm quality, reactive oxygen species, sperm motility, sperm viability

## Abstract

**Background and Aim:**

Cryopreservation is commonly used in canine reproductive biotechnology to preserve genetic material for long-term storage. However, the freeze–thaw process induces oxidative stress, mitochondrial dysfunction, and structural damage in spermatozoa, thereby reducing post-thaw sperm quality and fertility potential. Mitoquinone (MitoQ), a mitochondria-targeted antioxidant, has been suggested as a promising cryoprotective additive that mitigates mitochondrial reactive oxygen species and enhances cellular integrity. This study aimed to assess the dose-dependent effects of MitoQ supplementation on mitochondrial function, oxidative stress, and the post-thaw quality of cryopreserved canine spermatozoa to identify an optimal concentration for semen cryopreservation.

**Materials and Methods:**

Semen samples were collected from 10 healthy and fertile male dogs of various breeds. Each ejaculate was divided into four equal parts and cryopreserved with a Tris–fructose–egg yolk extender supplemented with different MitoQ concentrations (0, 100, 200, or 400 nM). After four weeks of storage in liquid nitrogen, the samples were thawed and evaluated *in vitro*. Post-thaw sperm quality was measured using computer-assisted sperm analysis to assess motility and kinematic parameters. Additional evaluations included sperm viability, plasma membrane integrity, acrosome integrity, lipid peroxidation levels, apoptotic status via flow cytometry, mitochondrial membrane potential, and intracellular hydrogen peroxide production. Data were analyzed using a mixed-effects model with dog as a random effect, and significance was set at p < 0.05.

**Results:**

MitoQ supplementation had concentration-dependent effects on several sperm quality parameters. The 200 nM MitoQ group showed the most consistent improvements in post-thaw sperm quality. Total motility and curvilinear velocity were significantly higher at this concentration compared to the control and other treatment groups. Additionally, sperm treated with 200 nM MitoQ displayed improved plasma membrane and acrosome integrity, reduced lipid peroxidation, and a lower proportion of dead sperm cells. Trends toward increased viability and enhanced mitochondrial activity were also seen at this concentration. In contrast, 400 nM MitoQ supplementation was linked to decreased membrane integrity and increased oxidative stress markers, suggesting possible pro-oxidant effects at higher doses. Overall, the results indicate that moderate MitoQ supplementation supports mitochondrial redox balance and partially reduces cryo-induced damage in canine spermatozoa.

**Conclusion:**

MitoQ supplementation at 200 nM modestly but consistently enhanced several *in vitro* indicators of post-thaw sperm quality in dogs, such as motility, membrane stability, and oxidative stress markers. These findings highlight the narrow therapeutic window of mitochondria-targeted antioxidants and stress the importance of dose optimization during semen cryopreservation. Although the observed improvements suggest potential benefits for reproductive biotechnology, further research with fertility-related functional outcomes and larger sample sizes is needed to confirm the practical reproductive impact of MitoQ supplementation in canine semen cryopreservation.

## INTRODUCTION

Sperm cryopreservation, a widely used method for the long-term preservation of male genetic material, plays an essential role in reproductive technologies such as artificial insemination [1–3]. Despite its benefits, about 50% of sperm sustain damage during cryopreservation, leading to a significant reduction in fertilizing ability compared with fresh sperm [4]. This damage appears as structural and functional impairments in vital molecular components, including adenosine triphosphate (ATP)-releasing nucleotides, lipids, and proteins [5–7], ultimately affecting the viability and functionality of thawed sperm.

Although sperm cryopreservation offers many benefits, current procedures can cause ultrastructural, biochemical, and functional changes in spermatozoa [8]. These harmful alterations result from a complex series of events involving ice crystal formation, mechanical stress, oxidative stress, freeze dehydration, and ice recrystallization during the freeze–thaw process [9–12]. Over the past decade, various cryoprotective agents, antioxidants, and proteins have been added to semen-freezing extenders to mitigate these effects. These additives aim to preserve membrane fluidity and permeability, regulate reactive oxygen species (ROS) production, and maintain acrosome integrity and mitochondrial function following cryopreservation [13–15].

Oxidative stress is a key factor in cryodamage to sperm cells, mainly impacting the sperm membrane and mitochondrial membrane potential. Mitochondria are a central source of endogenous ROS and are crucial for sperm energy metabolism [7]. Proper mitochondrial function is associated with improved sperm motility and viability, whereas mitochondrial dysfunction is linked to increased DNA fragmentation and reduced fertilizing capacity [16]. Therefore, many studies have explored the use of external antioxidants in semen extenders to control ROS levels through various oxidative pathways [17–19]. However, most conventional antioxidants can cross the outer mitochondrial membrane but cannot penetrate the inner mitochondrial membrane, which limits their effectiveness at the main site of ROS production [20].

To address this limitation, mitochondria-targeted antioxidants have been designed to deliver antioxidant molecules directly to the inner mitochondrial membrane, thereby increasing antioxidant levels at the main site of ROS production [21]. One such compound is mitoquinone (MitoQ), a mitochondria-targeted antioxidant made up of a tetraphenylphosphonium (TPP) cation linked to a ubiquinone moiety [19]. The positive charge of the TPP group allows MitoQ to accumulate selectively within the mitochondria, where it is reduced by the respiratory chain to its active antioxidant form, ubiquinol [24, 25]. This feature enables MitoQ to function as a chain-breaking antioxidant, capable of neutralizing mitochondrial ROS and shielding cellular components from oxidative damage.

Previous studies have shown that MitoQ enhances post-thaw viability and decreases lipid peroxidation in yellow catfish spermatozoa, and it prevents experimentally induced testicular degeneration in mouse models [26]. Additionally, oxidative stress has a complex role in embryonic development, affecting blastocyst formation depending on its intensity and duration. Therefore, controlled modulation of oxidative stress has been investigated to improve reproductive outcomes [27]. MitoQ supplementation in embryo culture media has been shown to reduce oxidative stress, prevent mitochondrial uncoupling, enhance blastocyst development, and decrease apoptosis in embryos derived from metabolically compromised oocytes [27].

Pharmacokinetic studies have also shown that MitoQ can reach testicular tissues after oral administration, effectively targeting mitochondria in Sertoli cells and providing a mechanistic basis for its potential role in enhancing sperm health and cryopreservation outcomes [28]. Furthermore, MitoQ-containing TPP has been reported to improve sperm quality and boost the activity of antioxidant enzymes, including superoxide dismutase, catalase, and glutathione peroxidase, in human sperm [29].

Despite growing interest in mitochondria-targeted antioxidants to improve sperm cryosurvival, their use in canine semen cryopreservation remains poorly understood. Several studies across different species have shown that mitochondria-targeted antioxidants can enhance sperm quality by improving mitochondrial function and decreasing oxidative stress during the freeze–thaw process [30–33]. Additionally, research involving the mitochondria-targeted antioxidant Mito-TEMPO has reported improvements in sperm quality and antioxidant enzyme activity in thawed human sperm, indicating that targeted mitochondrial redox regulation may be a promising strategy for protecting sperm cells from cryo-induced oxidative damage [34, 35]. However, the effectiveness of such mitochondria-focused antioxidant strategies in canine sperm has received limited research. Canine sperm exhibit high mitochondrial activity and are particularly vulnerable to oxidative stress during cryopreservation, which can impair motility, destabilize membranes, and increase apoptosis. In our previous study, supplementing canine semen extenders with 200 nM MitoQ combined with antifreeze protein III (AFP III) improved plasma membrane integrity and significantly decreased acrosomal damage in frozen–thawed sperm [36]. However, when MitoQ was used alone at the same concentration, no significant overall improvements in sperm quality were observed, suggesting that the cryoprotective effects of MitoQ may depend on its concentration or interactions with other cryoprotective agents. Therefore, a systematic assessment of how different doses of MitoQ alone affect post-thaw canine sperm quality is still needed, and the optimal concentration to enhance mitochondrial function and reduce oxidative stress in cryopreserved canine semen has yet to be definitively determined.

Based on this knowledge gap, the present study aimed to thoroughly evaluate how varying concentrations of the mitochondria-targeted antioxidant MitoQ influence the post-thaw quality of canine spermatozoa. Specifically, this research investigated whether adding different levels of MitoQ to semen extenders could improve key functional parameters of cryopreserved canine sperm. To accomplish this, semen samples were supplemented with increasing concentrations of MitoQ, cryopreserved, and then subjected to detailed *in vitro* analysis of sperm quality. The parameters assessed included sperm motility and kinematic traits via computer-assisted sperm analysis, viability, plasma membrane integrity, acrosome integrity, lipid peroxidation levels, apoptotic status, mitochondrial membrane potential, and intracellular hydrogen peroxide production. By combining insights into mitochondrial function and oxidative stress with traditional sperm quality indicators, this study aimed to identify the MitoQ concentration that offers the best cryoprotective benefit. Ultimately, the results are expected to help optimize antioxidant strategies for canine semen cryopreservation and deepen understanding of the role mitochondria-targeted antioxidants play in reproductive biotechnology.

## MATERIALS AND METHODS

### Ethical approval

All procedures involving animals in this study were conducted in strict accordance with internationally accepted guidelines for the ethical use of animals in scientific research. The experimental protocol complied with the Guidance on the Operation of the Animals (Scientific Procedures) Act 1986, the European Union Directive 2010/63/EU on the protection of animals used for scientific purposes, and the National Institutes of Health (NIH) Guide for the Care and Use of Laboratory Animals. Ethical approval for the use of animals and semen samples was obtained from the Animal Welfare Office of Justus-Liebig-University Giessen, Germany, under approval number kTV 11–2018, which was renewed on October 01, 2023.

All dogs included in the study were privately owned animals presented for routine reproductive evaluation. Only clinically healthy and fertile male dogs were enrolled after a comprehensive clinical examination to ensure normal reproductive health and the absence of systemic disease. Semen collection was performed using manual stimulation, a non-invasive, routinely applied procedure in canine reproductive medicine that does not cause pain or distress to the animals. No surgical procedures, pharmacological interventions, or experimental manipulations were applied to the animals during semen collection.

Written informed consent was obtained from all dog owners before participation in the study. Owners were informed about the purpose of the research, the procedures involved, and the intended scientific use of the collected semen samples. All procedures were conducted by trained veterinarians under controlled clinical conditions to ensure animal welfare and minimize stress during handling and semen collection.

The study design followed the principles of the 3Rs (Replacement, Reduction, and Refinement) to ensure responsible animal use in research. The number of animals was kept to the minimum required to obtain scientifically valid results, and all experimental procedures were conducted in accordance with standard veterinary practices to ensure the highest level of animal welfare throughout the study.

### Study period and location

The experimental work took place at the Clinic for Reproductive Medicine and Neonatology at Justus-Liebig University of Giessen, Germany, from November 15, 2023, to February 20, 2024.

### Semen collection and experimental design

Semen samples were collected from healthy, fertile male dogs to evaluate their breeding suitability. Ejaculates were obtained by manual stimulation from 10 clinically healthy, fertile male dogs (mean age: 5.0 ± 2.26 years), representing 8 different breeds. Two breeds, Collie and German Wirehaired Pointer, were represented by two dogs each, while the others, Cane Corso Italiano, English Bulldog, Whippet, Jack Russell Terrier, Great Dane, and Hovawart, were each represented by one dog. This diverse-breed sampling was intentionally chosen to reduce the influence of individual-dog effects and prevent breed-specific bias, not to compare specific breeds. All dogs underwent a clinical examination before semen collection and were only included if they had normal testicular morphology, normal body temperature, and no apparent systemic or reproductive issues. Semen was collected at room temperature (20°C–22°C) in a quiet setting, following established guidelines [37]. Ejaculates were obtained via manual stimulation with an estrous teaser bitch or a female phantom present. Three different ejaculate fractions were collected into sterile, prewarmed glass containers. One ejaculate per dog was used for the study, and only the second sperm-rich fraction was selected for further analysis.

After collection, the semen was carefully kept in a water bath at 37°C during the initial phase of the experiment. Next, each ejaculate was evaluated individually; samples showing >70%–75% progressive motility and normal morphology were selected for inclusion in this study and subsequent cryopreservation. Following the initial semen evaluation, each ejaculate (experimental unit) was divided into four equal aliquots, which were then randomly assigned to different experimental treatments. All aliquots were diluted with a Tris–fructose–egg yolk extender, prepared by dissolving 3.025 g of Tris (hydroxymethylaminomethane), 1.7 g of citric acid, and 1.25 g of fructose in 100 mL of double-distilled water. The solution pH was adjusted to 7.0, and the osmolality was set to 320 mOsm/kg. The extender was then supplemented with fresh, filtered chicken egg yolk (20% (v/v)), and glycerol was added in a single step to reach a final concentration of 5.0% (v/v) at 37°C during the dilution process [38].

Dilution was performed to achieve a final sperm concentration of 50–60 × 10^6^ sperm/mL. MitoQ (BYTORB1105367, Biorbyt Ltd., Cambridge, UK) was administered to the treatment groups at final concentrations of 100 nM, 200 nM, or 400 nM, while the control group received an equal volume of vehicle solution without the supplement. MitoQ was initially supplied as a 200 mg/mL solution in a 1:1 ethanol–water mixture (v/v), corresponding to an approximate stock concentration of 295 mM, based on its molecular weight of 678.82 g/mol. Upon delivery, the stock solution was aliquoted to reduce freeze–thaw cycles, stored at −80°C under light-protected conditions, and used within 3 months. Working solutions at millimolar concentrations (1–50 mM) were prepared by diluting the stock solution with an ethanol–water mixture (1:1, v/v) according to the manufacturer’s instructions. These intermediate solutions were further diluted into the experimental extender to achieve the desired final concentrations. All groups, including the control, contained the same final solvent concentration to ensure uniform conditions. The study included a control group and multiple MitoQ-supplemented treatment groups, each replicated 10 times.

The diluted samples were loaded into 0.25-mL straws [39] and sealed. Samples were cooled from 20°C–22°C to 5°C over 1.5 h using passive cooling in a refrigerator, without active temperature control. Following equilibration, the straws were frozen in liquid nitrogen (LN_2_) vapor, positioned 4–5 cm above the liquid nitrogen surface on a rack for 10 min in a standard LN_2_ container, and then plunged into liquid nitrogen. Frozen straws were stored in LN_2_, for 4 weeks. Straws were thawed individually in a 37°C water bath for 1 min for evaluation. After thawing, the samples were immediately processed for downstream analyses. Samples were maintained at 37 °C during post-thaw handling and assessments, and all assays were initiated within a consistent time frame following thawing.

### Sperm motility and velocity parameter assay

Sperm motility characteristics were assessed using a computer-aided sperm analysis system (CASA; AndroVision™, Minitüb GmbH, Tiefenbach, Germany) following the manufacturer’s guidelines for canine semen evaluation. The image sequences were recorded at 25 frames per second. The CASA output included total motility (TM) [%], progressive motility (PM) [%], average path velocity (VAP) [μm/s], straight-line velocity (VSL) [μm/s], curvilinear velocity (VCL) [μm/s], straightness (STR) [%], linearity (LIN) [%], amplitude of lateral head displacement (ALH) [μm], beat cross frequency (BCF) [Hz], and wobble (WOB) [%]. For each replicate, 5 μL of semen was placed in a prewarmed chamber slide (20-μm depth) and covered with a glass coverslip. Thresholds for TM and PM, as well as particle size and brightness parameters, were set according to the default canine-specific configuration provided by the AndroVision™ software. A minimum of 10 microscopic fields was examined to ensure that at least 200 spermatozoa were analyzed per sample. All CASA measurements were performed by a single trained operator who was blinded to the experimental treatments.

### Viability and lipid peroxidation assay

The percentage of viable sperm was determined using a modified eosin-B staining method (eosin-B, Merck KGaA, Darmstadt, Germany; sodium citrate, Merck KGaA) [40]. For each sample, 5 μL of diluted sperm was mixed with 10 μL of eosin solution on a prewarmed slide (final ratio 1:2) and allowed to dry at 25°C. Duplicate smears were prepared for each sample, and a single operator, blinded to the treatment groups, evaluated the slides. Under a 400× magnification microscope (Olympus, Japan), sperm with pink or red-stained heads were classified as dead, while those with unstained (white) heads were classified as viable. A total of 200 spermatozoa were counted per sample, and the results were expressed as the percentage of viable sperm.

MDA levels in sperm samples were measured as an indicator of lipid peroxidation (LPO) using the thiobarbituric acid (TBA) reaction method [41]. For each assay, 1 mL of the prepared sperm suspension was mixed with 2 mL of a TCA-TBA-HCl solution containing thiobarbituric acid, trichloroacetic acid (Merck KGaA), and 0.25 N hydrochloric acid. The mixture was boiled in a water bath at 100°C for 15 min, then cooled to 20°C–22°C. After centrifugation at 1200× g for 15 min, the supernatant’s absorbance was measured at 535 nm using a Hitachi U-2001 spectrophotometer (Hitachi Ltd., Tokyo, Japan). MDA levels were calculated using the specific extinction coefficient (1.56 × 10^5^ L/mol/cm) instead of a standard curve and were expressed as nmol per mL of semen. Blanks containing only the TCA-TBA-HCl reagent were included to correct for background absorbance.

### Membrane status and acrosome integrity

Sperm plasma membrane integrity was assessed using the hypo-osmotic swelling test (HOST). The hypo-osmotic solution was prepared to 100 mOsm, as previously described [42]. For the assay, 5 μL of sperm suspension was mixed with 50 μL of hypo-osmotic solution and incubated at 37°C for 30 min. Two drops of the mixture were placed on a prewarmed slide and covered with a coverslip after incubation. Positive HOST reactions were scored based on tail swelling: sperm with coiled or swollen tails were considered intact, while those with straight tails were considered damaged. At least 200 spermatozoa were evaluated per sample under a light microscope at 400× magnification. All slides were assessed by a single trained operator blinded to the treatment groups.

The acrosome integrity was assessed using a modified formalin–citrate solution (containing formaldehyde and sodium citrate, Merck KGaA) [43]. In this method, 10 μL of semen was mixed with 100 μL of formalin–citrate solution and placed on a microscope slide beneath a coverslip. A portion of the mixture was examined using a phase-contrast microscope (Hund Wetzlar H500, Helmut Hund GmbH, Wetzlar, Germany) equipped with a 100× oil immersion objective and a phase-contrast condenser, under Koehler illumination at 1000× magnification. Spermatozoa were classified as having intact acrosomes if the acrosomal cap appeared smooth, uniform, and clearly outlined, while swollen, irregular, or missing acrosomes were considered damaged. At least 200 sperm were counted from a minimum of three different microscopic fields for each sample. Results are expressed as the percentage of sperm with intact acrosomes.

### Analysis of apoptotic parameters

The percentage of apoptotic sperm was determined by flow cytometry using phosphatidylserine translocation as a marker. Initially, the sperm were washed twice with cold BioLegend cell staining buffer (Cat. No. 420201, BioLegend Inc., San Diego, CA, USA) and resuspended in annexin V-binding buffer (Cat. No. 422201, BioLegend Inc.) at a concentration of 1 × 10^6^ cells/mL. Subsequently, a 100 μL aliquot was incubated with 5 μL FITC-Annexin V (Cat. No. 640905, BioLegend Inc.) for 15 min at 20°C–22°C in the dark, followed by an additional incubation with 5 μL propidium iodide (PI, Cat. No. 421301, BioLegend Inc.) for 15 min. Before analysis, samples were diluted with 400 μL binding buffer, and at least 10,000 events were acquired per sample.

Flow cytometry was conducted on a CytoFLEX flow cytometer (BG49443, Beckman Coulter, Krefeld, Germany) at a low flow rate. To maintain consistent performance, the instrument was calibrated daily with standardized fluorescent beads. Fluorescence compensation controls were prepared using unstained, single-stained FITC, and single-stained PI samples to correct for spectral overlap between fluorophores. FITC was excited at 488 nm and measured on the 525/40 nm channel, while PI was excited at 561 nm and measured on the 610/20 nm channel. All data were acquired and analyzed with CytExpert software (Version 2.7 and Manufacturer: Beckman Coulter GmbH, Krefeld, Germany). Gating was systematically performed to identify distinct sperm subpopulations. Forward scatter (FSC) and side scatter (SSC) were initially used to gate singlet sperm populations, excluding debris and aggregates. The fluorescence intensity was then analyzed to classify cells into three categories: viable sperm (Annexin−/PI−), early apoptotic sperm (Annexin+/PI−), and late apoptotic or dead sperm (PI+). This strategy enabled accurate quantification of apoptotic cells across all treatment groups. Additionally, intracellular ROS levels were measured using DCFH-DA (Sigma-Aldrich, St. Louis, MO, USA) at a final concentration of 10 μM in 25 μL of buffer. Appropriate unstained and single-stained controls were included to establish baseline fluorescence and ensure accurate interpretation of ROS signals.

### Mitochondrial membrane potential and hydrogen peroxide levels

Rhodamine 123 (R123; 83702, Sigma-Aldrich, St. Louis, MO, USA) and PI (0.01 mg/ml, 537060, Sigma-Aldrich) were used to assess mitochondrial activity [31]. The initial semen sample, 300 μl, was treated with 10 μl of Rh123 (0.01 mg/ml) and incubated for 20 min at 20°C–22°C in the dark. The semen samples were then centrifuged at 500 g for 3 min and resuspended in 500 μl of Tris buffer. Finally, 10 μl of PI (1 μg/ml) was added before flow cytometry analysis, where sperm cells that showed a positive signal for Rh123 and a negative signal for PI were counted as having active mitochondria.

To measure hydrogen peroxide (H_2_O_2_) levels in sperm, semen samples were diluted with phosphate-buffered saline (PBS; Sigma-Aldrich) to a concentration of 5 × 10^6^ sperm/mL. Then, 25 μL of the fluorescent dye 2′,7′-dichlorofluorescein diacetate (Sigma-Aldrich) was added to 1 ml of the sperm suspension, and the mixture was incubated at 20°C–22°C for 40 min. After centrifugation to remove the supernatant, the sperm pellet was resuspended in PBS to the same concentration and incubated at 20°C–22°C for 10 min. Before flow cytometric analysis, a 2 μL addition of PI (1 μg/mL) was added to the semen samples [31].

### Statistical analysis

Data normality was assessed using the Shapiro-Wilk test, confirming that all variables were normally distributed (p > 0.05). Statistical analyses were conducted with the General Linear Model procedure in SAS (version 9.1; SAS Institute Inc., Cary, NC, USA). To account for repeated measures from multiple ejaculates per dog, a dog was included as a random effect in a mixed model. The model was specified as: Yij = μ + Ti + Dj + Eij, where Yij is the observed value, μ is the overall mean, Ti is the fixed effect of treatment, Dj is the random effect of dog, and Eij is the residual error. Orthogonal contrasts were used for mean comparisons, including comparisons of each MitoQ concentration versus the control, and linear and quadratic trends across concentrations were evaluated. Multiple comparisons were handled with the Tukey-Kramer adjustment. Statistical significance was set at p < 0.05, and results are shown as mean ± standard deviation (Mean ± SD).

## RESULTS

### Data normality assessment

The normality of all data was evaluated using statistical tests, and all parameters adhered to a normal distribution (p > 0.05). Normality was confirmed for the following primary outcomes: TM (p = 0.72), membrane integrity (p = 0.49), PM (p = 0.56), curvilinear velocity (VCL, p = 0.68), average path velocity (VAP, p = 0.61), and straight-line velocity (VSL, p = 0.59). Secondary outcomes, including early and late apoptosis (p = 0.66 and 0.57, respectively), lipid peroxidation (LPO, p = 0.62), acrosome integrity (p = 0.54), viability (p = 0.58), and intracellular ROS-H_2_O_2_ (p = 0.68), also followed a normal distribution, supporting the use of parametric statistical methods.

### Effects of MitoQ on sperm kinematic parameters

Table 1 summarizes the effects of different concentrations of MitoQ (100, 200, and 400 nM) on the primary outcomes TM and membrane integrity, as well as on secondary outcomes. TM was significantly higher in the 200 nM MitoQ group (38.40 ± 2.94%, p < 0.05) compared to the control group (33.30 ± 2.63%), the 100 nM group (33.71 ± 2.82%), and the 400 nM group (34.55 ± 3.35%).

**Table 1:** Kinematic parameters of cryopreserved canine sperm supplemented with MitoQ (Means ± Standard deviation).

CASA parameters	Control	100 nM	200 nM	400 nM
TM [%]	33.30 ± 2.63^b^	33.71 ± 2.82^b^	38.40 ± 2.94^a^	34.55 ± 3.35^b^
PM [%]	32.79 ± 2.45	28.74 ± 2.34	33.34 ± 2.71	28.75 ± 2.97
VAP [μm/s]	22.91 ± 1.52	23.19 ± 1.35	26.64 ± 0.96	22.59 ± 1.57
VSL [μm/s]	20.89 ± 1.16	18.82 ± 1.03	18.74 ± 0.82	18.21 ± 1.25
VCL [μm/s]	37.67 ± 1.82^b^	37.06 ± 2.00^b^	43.73 ± 2.55^a^	37.63 ± 2.44^b^
STR [%]	0.45 ± 0.03	0.42 ± 0.25	0.43 ± 0.02	0.42 ± 0.02
LIN [%]	0.79 ± 9.06	0.81 ± 0.01	0.82 ± 0.01	0.81 ± 0.01
ALH [μm]	0.51 ± 0.02	0.50 ± 0.02	0.50 ± 0.02	0.49 ± 0.02
BCF [Hz]	5.38 ± 0.51	4.71 ± 0.44	4.94 ± 0.45	4.53 ± 0.45
WOB [%]	0.62 ± 0.02	0.61 ± 0.02	0.61 ± 0.09	0.60 ± 0.02

ALH = Amplitude of lateral head displacement, BCF = Beat cross frequency, LIN = Linearity, PM = Progressive motility, STR = Straightness, TM = Total motility, VAP = Average path velocity, VCL = Curvilinear velocity, VSL = Straight linear velocity, and WOB = Wobble. Different letters indicate statistically significant differences (*p* < 0.05), (n = 10).

PM showed a tendency to improve at 200 nM (33.34 ± 2.71%, p > 0.05), surpassing the values in the 100 nM (28.74 ± 2.34%) and 400 nM (28.75 ± 2.97%) groups, while remaining comparable to the control group (32.79 ± 2.45%).

Among the velocity parameters, curvilinear velocity (VCL) was significantly higher at 200 nM (41.73 ± 2.55 μm/s, p < 0.05) compared to the control (37.67 ± 1.82 μm/s), 100 nM (37.06 ± 2.08 μm/s), and 400 nM (37.63 ± 2.44 μm/s) groups. In contrast, average path velocity (VAP) and straight-line velocity (VSL) showed no significant differences among the groups (p > 0.05). Similarly, other CASA parameters, including straightness (STR), linearity (LIN), amplitude of lateral head displacement (ALH), BCF, and wobble (WOB), did not differ significantly (p > 0.05). Overall, TM, PM, and VCL tended to improve at 200 nM MitoQ, although the functional significance of these changes remains to be determined.

### Effects of MitoQ on sperm viability and lipid peroxidation

**Figure 1:**
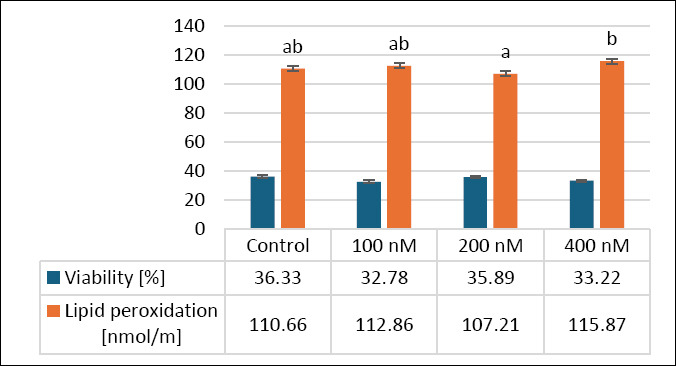
Effects of different MitoQ concentrations on viability and lipid peroxidation levels in frozen–thawed canine spermatozoa. Distinct letters signify statistically significant differences (p < 0.05), (n = 10).

Figure 1 shows how MitoQ supplementation in extenders affects secondary outcomes, specifically sperm viability and lipid peroxidation (LPO), in frozen–thawed canine semen. Concerning viability, there were no significant differences (*p* > 0.05) between the control group (36.33 ± 2.43%) and the treatment groups: 100 nM (32.78 ± 2.65%), 200 nM (35.89 ± 2.13%), and 400 nM MitoQ (33.22 ± 1.67%). A tendency toward improvement was noticed, with the 200 nM group showing the highest viability, although these differences were not statistically significant.

LPO levels in the control group (110.66 ± 8.65 nmol/mL) were similar to those in the 100 nM (112.86 ± 7.23 nmol/mL) and 400 nM (115.87 ± 5.71 nmol/mL) groups (p > 0.05). The 200 nM MitoQ group (107.21 ± 7.11 nmol/mL) showed a significant reduction in LPO compared to the 400 nM group (p < 0.05), while no significant difference was found between the control and 200 nM groups (p > 0.05). These results suggest that 200 nM MitoQ may slightly improve sperm viability and reduce oxidative stress, indicating its potential effects on secondary outcomes, including apoptosis, LPO, mitochondrial activity, and intracellular ROS-H_2_O_2_.

### Effects of MitoQ on membrane and acrosome integrity

**Figure 2:**
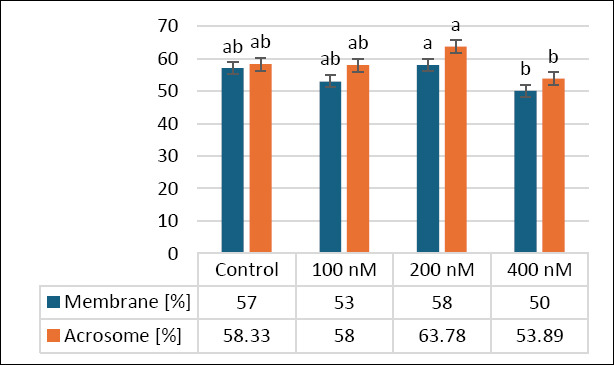
Effects of different MitoQ concentrations on membrane and acrosomal integrity in frozen–thawed canine spermatozoa. Distinct letters signify statistically significant differences (p < 0.05), (n = 10).

Figure 2 shows how different MitoQ levels (100, 200, and 400 nM) affect secondary outcomes, specifically membrane and acrosome integrity, in frozen–thawed canine sperm. The control group had an average membrane integrity of 57.00 ± 2.13%, with no significant differences between the 100 nM (53.33 ± 2.45%, p > 0.05) and 200 nM (58.00 ± 2.67%, p > 0.05) groups. However, the 400 nM group showed a significant decrease in membrane integrity (50.00 ± 2.31%) compared with the 200 nM group (p < 0.05), suggesting that the highest concentration may have harmful effects.

Regarding acrosome integrity, the control (58.33 ± 2.67%) and 100 nM (58.00 ± 2.45%) groups showed no significant difference from the 200 nM group (63.78 ± 2.71%, p > 0.05). The 400 nM group had the lowest acrosome integrity (53.89 ± 2.52%) and was significantly lower than the 200 nM group (p < 0.05). These results suggest that 200 nM MitoQ may help maintain membrane and acrosome integrity, whereas higher concentrations (400 nM) could adversely affect these outcomes, highlighting the need for dose optimization.

### Effects of MitoQ on sperm viability and apoptotic status

**Figure 3:**
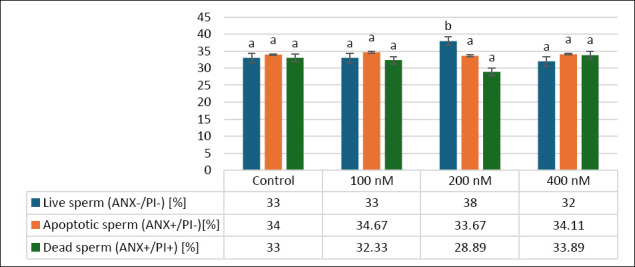
Effects of different MitoQ concentrations on live, apoptotic, and dead sperm in frozen–thawed canine spermatozoa. Distinct letters (a, b,…) indicate statistically significant differences (p < 0.05) (n = 10).

Figure 3 shows the effects of different MitoQ concentrations (100, 200, and 400 nM) on secondary outcomes related to sperm viability, including percentages of live, apoptotic, and dead sperm in frozen–thawed canine semen. The percentage of live sperm was similar between the control (33 ± 1.87%) and 100 nM (33 ± 2.13%) groups (p > 0.05), whereas the 200 nM group had the highest live sperm percentage (38 ± 3.13%). Although this difference was not statistically significant compared to the control or 100 nM groups, the 200 nM group was significantly higher than the 400 nM group (32 ± 2.35%, p < 0.05).

For apoptotic sperm, the 200 nM group showed a distinct pattern compared to the other groups: control (34 ± 2.82%), 100 nM (34.67 ± 1.98%), 200 nM (33 ± 2.22%), and 400 nM (34.11 ± 2.15%), as shown in Figure 3.

Regarding dead sperm, the control (33 ± 1.6%) and 100 nM (32.33 ± 2.54%, p > 0.05) groups were similar, while the 200 nM group had the lowest percentage (28.33 ± 2.69%), which was significantly lower than both the control (p < 0.05) and 400 nM (33.89 ± 1.75%, p < 0.05) groups. Overall, MitoQ at 200 nM tended to improve sperm viability by increasing the number of live sperm and decreasing the proportion of dead sperm, highlighting its potential benefit on secondary outcomes in frozen–thawed canine semen.

### Effects of MitoQ on mitochondrial activity and ROS-H_2_O_2_ levels

**Figure 4:**
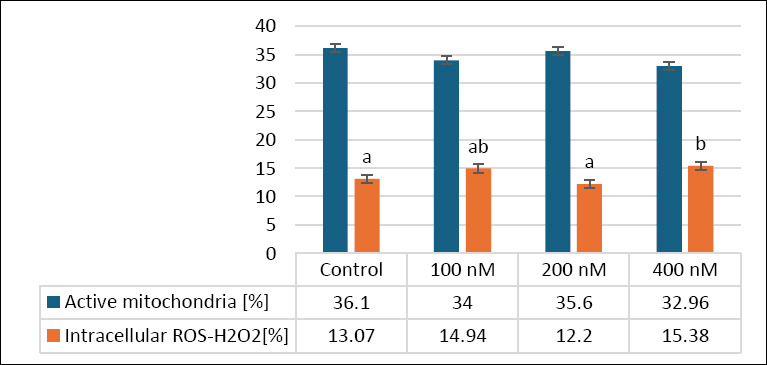
Effects of different MitoQ concentrations on mitochondrial activity and intracellular reactive hydrogen peroxide levels in frozen and thawed canine spermatozoa (n = 10).

Figure 4 illustrates the effects of various MitoQ concentrations (100, 200, and 400 nM) on secondary outcomes related to mitochondrial function and oxidative stress, specifically mitochondrial activity and intracellular ROS-H_2_O_2_ levels, in frozen–thawed canine spermatozoa. For mitochondrial activity, the control group exhibited the highest percentage (36.1 ± 1.53%), followed by the 200 nM group (35.6 ± 2.27%) and the 100 nM group (34 ± 2.67%). The 400 nM group showed the lowest mitochondrial activity (32.96 ± 1.29%). Despite these differences, statistical analysis revealed no significant differences among the groups (p > 0.05).

Regarding intracellular ROS-H_2_O_2_ levels, the control group recorded 33 ± 2.41%, similar to the 100 nM (33 ± 2.11%) and 400 nM (32 ± 1.72%) groups. The 200 nM group exhibited the highest ROS-H_2_O_2_ level (38 ± 2.01%), which was significantly higher than that in the 400 nM group (p < 0.05), although no significant differences were seen between the control and either the 100 or 200 nM groups (p > 0.05). These results indicate that MitoQ at 200 nM may slightly increase intracellular ROS-H_2_O_2_ without significantly impacting mitochondrial activity, emphasizing the importance of dose optimization to maintain oxidative balance in frozen–thawed canine sperm.

## DISCUSSION

### Cryopreservation-induced oxidative stress and mitochondrial vulnerability

Cryopreservation remains an essential method for long-term storage of genetic material in animal breeding and conservation efforts; however, it is often associated with damage to spermatozoa’s structure and function. These changes mainly result from cold shock, osmotic stress, and oxidative stress, which together damage plasma membrane integrity, acrosomal stability, and mitochondrial function [1, 2, 31, 36]. Mitochondria are especially vulnerable to cryo-induced injury because they are both a major source and target of ROS. Excessive mitochondrial ROS production during freezing and thawing can cause lipid peroxidation and DNA damage, and trigger apoptosis, ultimately decreasing sperm viability and function [31, 36]. As a result, there is ongoing interest in developing cryopreservation extenders containing antioxidants to protect mitochondrial function and reduce oxidative damage [1, 2, 31, 43].

Among mitochondria-targeted antioxidants, MitoQ has gained significant attention because it can specifically accumulate in the inner mitochondrial membrane and directly neutralize ROS, especially superoxide anions and hydrogen peroxide [31, 44–47]. Previous research in various species, including bovine, caprine, and avian models, has shown improvements in post-thaw sperm motility, membrane integrity, and acrosomal preservation after supplementing with low concentrations of MitoQ [30, 31, 44–47].

### Effects of MitoQ supplementation on post-thaw sperm quality

Supplementation with 200 nM MitoQ led to the most consistent improvements in post-thaw motility, acrosome integrity, and plasma membrane stability, along with a decrease in markers linked to cell death. However, it should be noted that ROS-H_2_O_2_ levels at 200 nM were not always lower than those at 400 nM, suggesting that oxidative protection at this concentration may not be solely reflected by a reduction in all ROS markers but rather by a balance between controlled ROS signaling and preventing excessive oxidative damage. Therefore, the improvements in sperm quality observed at 200 nM likely result from qualitative modulation of mitochondrial redox homeostasis rather than a uniform decrease in ROS production.

Although the overall increases in parameters such as TM and curvilinear velocity were small, these changes remain biologically significant in the context of cryopreserved semen, where post-thaw sperm quality is naturally compromised. Even modest improvements can greatly raise the proportion of sperm that are functionally competent. From a practical perspective, slight enhancements in motility and velocity could improve sperm transport, interaction with the zona pellucida, and fertilization success, especially when only a subset of sperm survives cryopreservation. However, because fertilization capability, pregnancy outcomes, and embryo development were not evaluated, any conclusions about whether these *in vitro* improvements translate to *in vivo* fertility should be drawn with caution.

These findings support the hypothesis that MitoQ can effectively reduce oxidative and structural damage caused by cryopreservation when used at an optimal concentration.

### Comparison with previous studies and possible biological explanations

Notably, the beneficial effects observed at 200 nM MitoQ differ from our previous findings, where MitoQ supplementation alone at the same concentration did not lead to a statistically significant improvement in cryopreserved canine sperm quality. Beneficial outcomes were only seen when MitoQ was combined with AFP III. This discrepancy highlights that statistical significance alone does not fully capture biological efficacy and underscores the importance of considering modest yet consistent improvements within the broader physiological context of sperm cryotolerance.

The divergence further indicates that the biological effect of MitoQ depends on multiple interacting factors, not just concentration alone. Variations in donor populations may play a significant role. Including multiple breeds with different seminal traits can greatly influence mitochondrial redox balance and the response to mitochondria-targeted antioxidants. Breed-related differences in membrane lipid composition, cholesterol-to-phospholipid ratios, endogenous antioxidant defenses, and seminal plasma composition affect susceptibility to oxidative stress and cryo-induced membrane destabilization [16, 31, 36]. These inherent differences may determine not only whether statistical significance is achieved but also whether the observed changes result in biologically meaningful improvements in sperm function.

Methodological differences between studies, such as subtle variations in extender composition, freezing rate, or storage conditions, may also affect MitoQ stability and bioavailability during cryopreservation.

### Concentration-dependent effects of MitoQ

The concentration-dependent dual activity of MitoQ is an important and biologically relevant factor to consider. At low concentrations, MitoQ functions as an effective mitochondrial antioxidant; however, at higher doses, it may engage in redox cycling and paradoxically increase ROS production [44–47]. Supplementing with 400 nM MitoQ was associated with higher levels of oxidative stress markers, consistent with previously reported biphasic effects of MitoQ in spermatozoa and somatic cells [31, 44–47]. Nonetheless, its role in activating adenylate kinase 2 in the sperm flagellum further clarifies its ability to improve motility parameters, while enhanced mitochondrial function and cell survival have been repeatedly observed across different species, supporting MitoQ’s effectiveness as a targeted mitochondrial antioxidant for semen preservation [48–50].

Although some of these differences reached statistical significance, their biological importance mainly lies in identifying a threshold beyond which mitochondrial protection diminishes and oxidative damage worsens, rather than in any functional advantage. This narrow therapeutic window underscores the need for precise dose optimization.

### Mechanistic implications of mitochondrial protection

From a mechanistic perspective, the improvements observed at 200 nM MitoQ are likely linked to stabilization of mitochondrial membrane potential, protection of ATP production, and inhibition of apoptosis-related pathways. Sperm mitochondria control critical functions, including energy metabolism and calcium homeostasis, which are vital for motility and fertility [16, 31, 36]. Cryo-induced mitochondrial dysfunction promotes cytochrome c release and caspase activation, leading to apoptotic cell death and decreased sperm viability [36, 51–54].

The reduction in dead sperm and the trend toward improved mitochondrial activity at 200 nM indicate meaningful preservation of mitochondrial integrity, although statistical significance was not consistently observed across all endpoints. Overall, these results support the use of MitoQ as a mitochondria-targeted antioxidant to enhance certain post-thaw sperm parameters in dogs at moderate concentrations.

The biological significance of these findings comes from the cumulative effect of several small but consistent improvements across multiple sperm quality parameters, rather than from isolated statistically significant results. However, the absence of fertility-related functional endpoints is a major limitation, so conclusions about cryoprotective effectiveness should be limited to improvements in *in vitro* sperm quality rather than extended to reproductive performance. Additionally, the variability in responses among individuals and across studies indicates that MitoQ’s effectiveness depends on context and is influenced by factors such as dosage, sperm source, and experimental conditions. Therefore, more research is needed to refine dosing strategies and to explore whether combining MitoQ with other cryoprotective agents can enhance and stabilize its beneficial effects.

### Limitations and future directions

This study has several limitations that should be considered when interpreting the results. The relatively small sample size may have reduced the statistical power to detect subtle treatment effects, and some biologically relevant trends may therefore not have reached statistical significance. The exclusive use of canine sperm limits broader applicability to other species, and the inclusion of multiple breeds may have introduced biological variability, obscuring breed-specific responses. Functional endpoints directly related to reproductive success, such as fertilization capacity or embryo development, were not evaluated; thus, the biological significance of the observed improvements must currently be viewed as indicative rather than conclusive.

Additionally, the effects of long-term cryostorage were not evaluated, and only some ROS markers were measured, limiting a full understanding of redox regulation. Intermediate MitoQ concentrations between 200 and 400 nM were not tested, restricting detailed dose–response analysis. Future research should include functional fertility tests, longer storage assessments, a wider range of oxidative stress markers, and more precise dose trials. These methods will be crucial for more clearly connecting statistically significant findings to biologically relevant reproductive outcomes.

## CONCLUSION

The present study showed that adding 200 nM MitoQ to the cryopreservation extender yielded the most consistent improvements across various post-thaw sperm quality parameters in canine semen. Specifically, this concentration was associated with higher TM and curvilinear velocity, better membrane and acrosome integrity, and a lower percentage of dead sperm after thawing. Additionally, the 200 nM treatment reduced lipid peroxidation compared with the higher concentration group, indicating a partial reduction in oxidative stress during the freeze–thaw process. Although mitochondrial activity and intracellular ROS-H_2_O_2_ levels did not consistently decrease across all treatments, the overall trend suggests that MitoQ at moderate concentrations may help maintain mitochondrial redox balance rather than simply suppress ROS production.

From a practical perspective, even small improvements in post-thaw motility and membrane integrity can be biologically significant in cryopreserved semen, where only a small portion of sperm remains functionally competent after thawing. Increasing the number of viable and motile sperm may enhance sperm transport, interaction with the zona pellucida, and the chances of successful fertilization in assisted reproductive technologies such as artificial insemination. Therefore, using mitochondria-targeted antioxidants like MitoQ in cryopreservation extenders offers a promising approach to improve semen preservation protocols in canine reproduction.

A key strength of this study is its comprehensive assessment of sperm quality, including kinematic parameters, membrane and acrosome integrity, apoptosis, mitochondrial activity, and oxidative stress markers. These combined factors offer a multidimensional view of sperm functional health after cryopreservation. Additionally, using a mitochondria-targeted antioxidant sheds light on the role of mitochondrial redox regulation in sperm cryotolerance.

In summary, the findings of this study suggest that MitoQ, when used at an appropriate concentration, can enhance certain post-thaw sperm quality parameters and may serve as a valuable addition to canine semen cryopreservation protocols. Further research combining both *in vitro* and *in vivo* fertility assessments will be necessary to fully determine its practical usefulness in reproductive biotechnology and conservation breeding programs.

## DATA AVAILABILITY

The supplementary data can be made available from the corresponding author upon request

## AUTHORS’ CONTRIBUTIONS

AF: Investigation, sampling, data analysis, sample collection, writing the original manuscript, and review and editing of the manuscript. ED: Sample collection, writing the original manuscript, and review and editing of the manuscript. AW: Writing the original manuscript, supervision, and review and editing of the manuscript. All authors have read and approved the final version of the manuscript.
